# Soybean Hydrophobic Protein Response to External Electric Field: A Molecular Modeling Approach

**DOI:** 10.3390/biom3010168

**Published:** 2013-02-11

**Authors:** Ashutosh Singh, Valérie Orsat, Vijaya Raghavan

**Affiliations:** Department of Bioresource Engineering, McGill University, 21,111 Rue Lakeshore, Ste-anne-de-bellevue, QC, H9X 3V9, Canada; E-Mails: valerie.orsat@mcgill.ca (V.O.); vijaya.raghavan@mcgill.ca (V.R.)

**Keywords:** molecular dynamic modeling, soybean hydrophobic protein, electromagnetic fields

## Abstract

The molecular dynamic (MD) modeling approach was applied to evaluate the effect of an external electric field on soybean hydrophobic protein and surface properties. Nominal electric field strengths of 0.002 V/nm and 0.004 V/nm had no major effect on the structure and surface properties of the protein isolate but the higher electric field strength of 3 V/nm significantly affected the protein conformation and solvent accessible surface area. The response of protein isolate to various external field stresses demonstrated that it is necessary to gain insight into protein dynamics under electromagnetic fields in order to be able to develop the techniques utilizing them for food processing and other biological applications.

## 1. Introduction

The field of molecular dynamic (MD) modeling is concerned with the atomic and molecular interactions that take place within the physical system that governs its microscopic and macroscopic behavior. It was developed as a tool to numerically simulate simplistic models in statistical mechanics [1], but advancement in computer technology paved the path for the development of MD modeling techniques to understand the structural and dynamic properties of biomolecules. The technique has been widely used in the field of molecular biology and pharmaceutical sciences, and it has lead to the development of a wide range of efficient and accurate drug systems based on the structural and dynamic data obtained from the simulation. However, its use in food process engineering has been negligible; its development in and application to modern sophisticated unit operations in food manufacturing is essential to evaluate the changes in the organoleptic and nutritional quality of the final food product. Current consumer preference is for high nutritional food products, manufactured through the application of energy efficient processes, which has challenged industries and researchers to develop and utilize newer techniques such as microwave or pulsed electric field and radio frequency, alone or in combination with traditional unit operations [2,3].

The development and optimization of food processes depends on mechanical limitations and the needs stated by the consumers in terms of the final product. In general, the quality of the final product is estimated by quantifying its components such as proteins, starch, mineral, vitamins and other constituents. However, the quality of the constituents themselves is rarely considered as a parameter for the optimization process. Application of unit operations such as drying, freezing, filtration, *etc*. not only negatively impacts the quantity of the food constituents but also have a significant effect on their quality. Proteins play a fundamental role for humans and are an important constituent of food. Processing methods and parameters have diverse effects on protein quality and quantity. Protein function depends on its structure and any change due to the presence of external stresses—such as heat, pH, chemicals and electromagnetic field—can lead to its denaturation leading to nonfunctional characteristics. The effect of an electromagnetic field on protein conformation has been extensively studied [4,5,6,7,8], but it is challenging to quantify the effect, as the alteration to conformation takes place in a very short time span (nanoscale) [9]. It is our understanding that in order to gain the insight into the mechanism of the interaction between electromagnetic fields and food constituents, it is necessary to understand the influence at atomistic and molecular level.

We have used a MD modeling approach to analyze the effect of a high electric field on the conformation of soybean hydrophobic protein (SHP), which is part of the soybean protein isolate. Soybean protein (SP) is one of the most extensively studied food proteins. Protein isolates from soybean are prepared from defatted soybean flour using a water extraction process under mild alkaline conditions [8]. SP has excellent functional and nutritional properties [10,11], but conventional thermal treatment processes used in food industries have undesirable effects on their solubility and water absorption characteristics [8,12]. Several novel processing techniques apply electromagnetic waves such as microwave and radio frequency or high electric field to achieve superior food quality [2]. In 2011, Xiang *et al.* [8] studied the structural modification induced by the application of PEF on soybean protein isolate. They reported that the application of an electric field intensity of 20–25 kV/cm and pulse number varying from 30–120 induced changes in the microenvironment of the soy protein’s tryptophan residues from a less polar to a more polar environment, they indicated that these changes in the polarity was brought by partial denaturation of the protein under PEF treatment. 

Hence in this study emphasis, has been put on quantification of conformational changes using root mean square deviation, radius of gyration and total dipole moment data and its effect on surface hydrophobicity and hydrophilicity of SHP.

## 2. Results and Discussion

### 2.1. Secondary Structure Analysis

**Figure 1:**
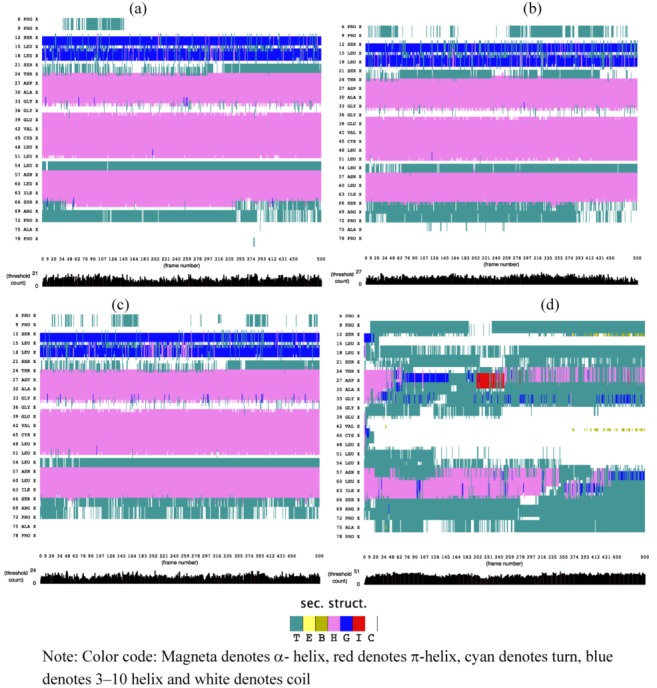
STRIDE showing evolution of secondary structure of soybean hydrophobic protein (SHP) (a) without electric field (b) 0.002 V/nm (c) 0.004 V/nm and (d) 3 V/nm external electric field.

The effect of an external electric field on secondary structures of SHP was evaluated using the STRIDE algorithm. Figure 1 demonstrates that application of electric field strengths 0.002 V/nm and 0.004 V/nm had no major effect on the helical region of SHP, but minor changes can be observed on turns and coils, as compared to no electric field. Application of a 3V/nm electric field severely disrupts the helical region of the protein, as compared to 0.002 and 0.004 V/nm. It is interesting to observe that the application of 3V/nm lead to a transition between a- helix and p-helix, and similar transitions were observed by Budi *et al*., (2005) [17] in their study on the effect of an electric field on Insulin protein Chain-B conformation. They stated that this transition is an integral part of protein’s dynamic property and plays an important role in defining its function [17]. 

### 2.2. Dipole Moment Distribution

Proteins, due to their secondary structure conformations such as helices, sheets, turns, coils, *etc*. possess electric dipole moment and application of external electric field induces an alignment change in them with respect to the direction of the applied field [18]. The electric dipole of a protein is represented as:

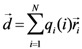
(Equation 1)


**Figure 2:**
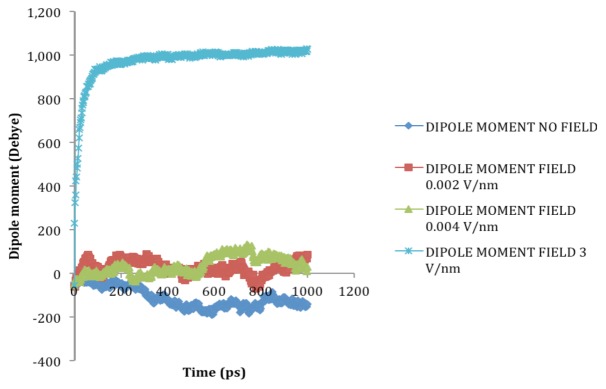
Total dipole moment of SHP under the influence of an external electric field.

**Figure 3:**
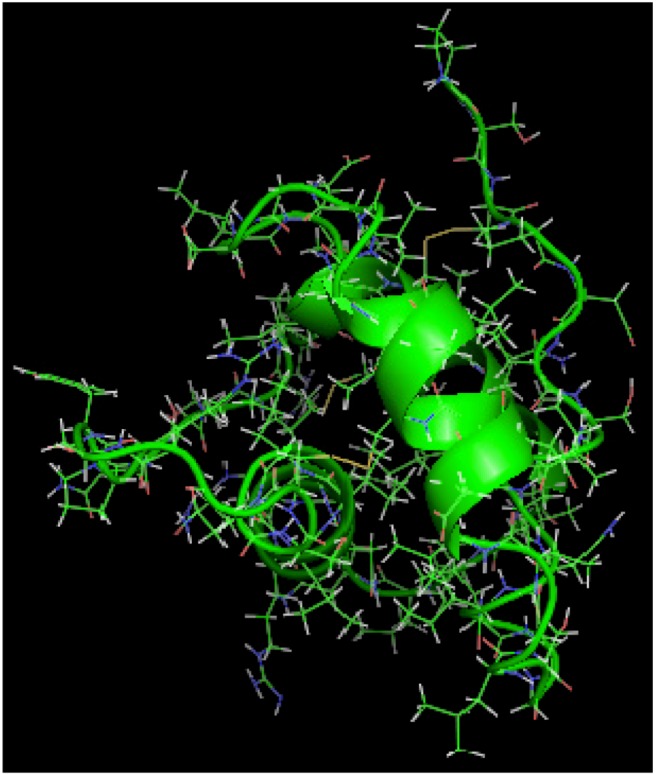
SHP protein under no external electric field.

**Figure 4:**
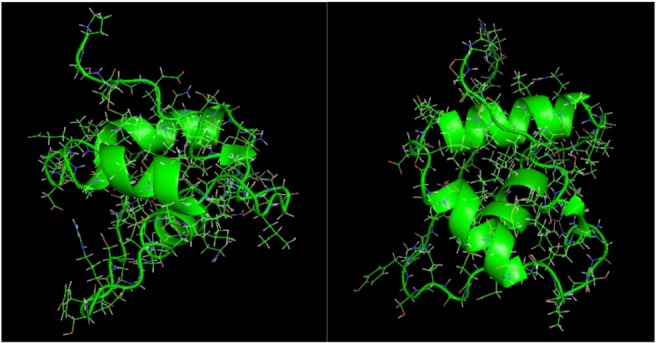
SHP protein snapshot at the end of the MD simulation under the influence of 0.002 V/nm and 0.004 V/nm.

**Figure 5:**
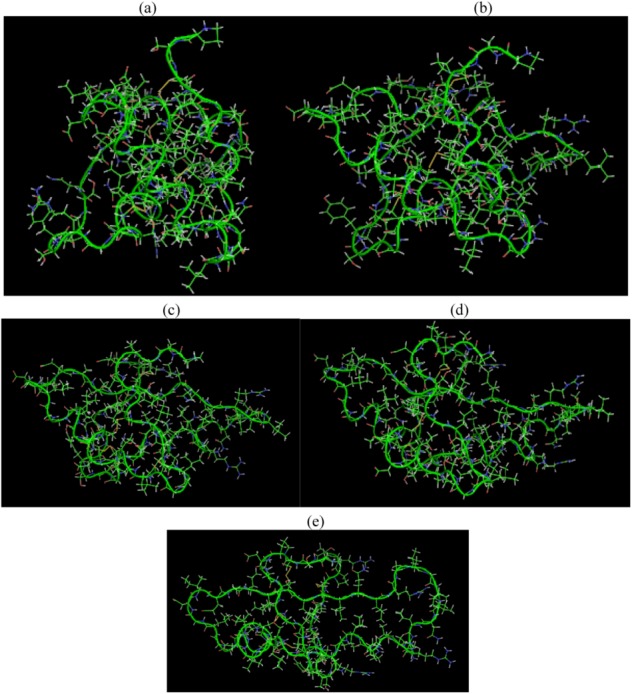
SHP snapshots at the beginning (a) and at different time spans (b), (c), (d) and at the end of the MD simulation (e) with electric field 3 V/nm.

Where 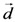 is the dipole, *q_i_* is the charge of the atom *i*, 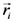 is the directional vector of each atom and *N* is the number of atoms. When an external electric field *E* is applied the protein orients itself in the direction of the field and in our study, the electric field was applied in the *x-axis*. Depending on the strength of the applied field, unfolding or re-orientation of protein is observed. From Figure 2 and Table 1 it can be noted that application of an external electric field changed the total dipole moment of SHP under MD simulation. The total dipole moment for applied field strength of 3V/nm increased until 1000 Debye in approximately 200 ps and remained constant thereafter because the protein completely unfolded and all the major secondary structures were lost (Figure 5). From Figure 3 and Figure 4 it can also be observed that at the end of the simulation, the helices of SPI were realigned in the direction of the applied electric field, but no preferential alignment of helices were observed in the reference (no field), confirming that under an external electric field, proteins orient themselves in the direction of the field [9,15,17]. 

**Table 1:** RMSD, Radius of Gyration and Total Dipole moment of the SHP protein Backbone, averaged over 1ns of simulation time.

Molecule	Electric field strength (V/nm)	RMSD average (nm)	Rg average (nm)	Total Dipole moment (Debye)
SHP (1HYP)	0	0.122 ± 0.022	1.150 ± 0.024	−121 ± 45.311
SHP (1HYP)	0.002	0.114 ± 0.016	1.163 ± 0.037	27 ± 31.444
SHP (1HYP)	0.004	0.119 ± 0.021	1.150 ± 0.021	38 ± 42.460
SHP (1HYP)	3	0.803 ± 0.060	1.435 ± 0.043	971 ± 102.666

### 2.3. Root Mean Square Deviation (RMSD):

The RMSD of a protein during simulation provides a quantitative measure of any structural variation. RMSD is calculated by comparing the structure of simulated protein during simulation with the reference [19]. RMSD of a protein is represented as:

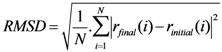
(Equation 2)


Where *r_final_*(*i*) is the final coordinates of an atom *i*, and *r_initial_*(*i*) is the initial coordinate of the atom *i*, and *N* is the number of atoms.

**Figure 6:**
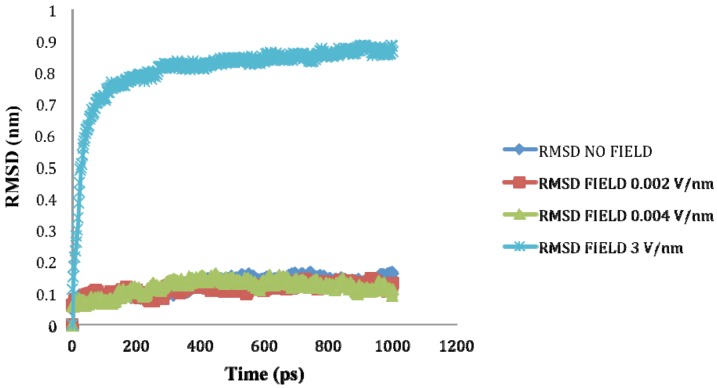
RMSD of SHP backbone under the effect of external electric field.

The average value of RMSD for 1ns simulation is shown in Table 1. It can be assessed from Table 1 and Figure 6 that as the application of an electric field strength of 0.002 V/nm and 0.004 V/nm had no major effect on the stability of the SPI structure as their RMSD value were similar to the reference (no field), but at 3V/nm the partial and complete loss of the helical secondary structures produced a higher RMSD value.

### 2.4. Radius of Gyration (Rg)

The Rg of the protein quantifies the distribution of the atoms in the space relative to their center of mass; it provides an understanding of the changes in shape and size of the protein under the influence of external stresses such as an electric field [17,20]. It can be calculated using Equation 3.

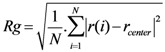
(Equation 3)


Where *r*(*i*) is the coordinates of an atom *i* and *r_center_* is the coordinates of the protein’s center of mass, *N* is the number of atoms.

**Figure 7:**
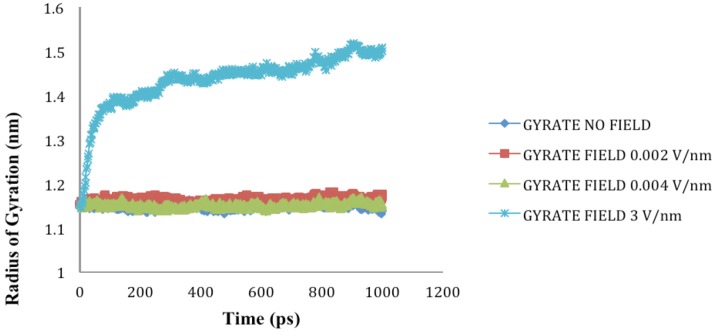
SHP radius of gyration (Rg) in the absence and presence of various external electric fields.

The radius of gyration (Rg) values for SHP under the influence of electric field strength 0.002 V/nm and 0.004 V/nm did not vary much with respect to the reference, because no significant change to the structure was observed, but as the electric field of 3 V/nm was applied the Rg value obtained was higher compared to the reference and other applied intensities because of the loss of secondary structures and resulting protein unfolding (Figure 7).

### 2.5. Solvent Accessible Surface Area

**Figure 8:**
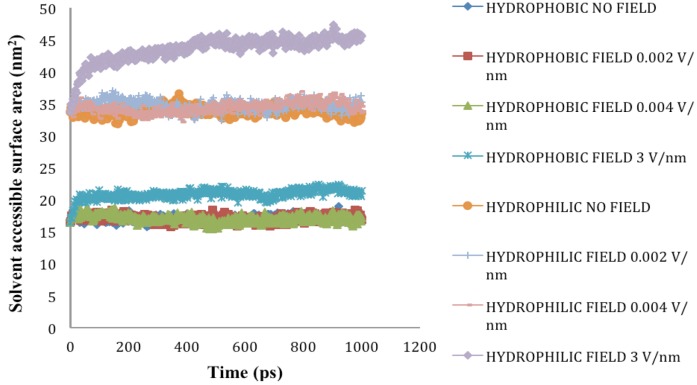
Solvent accessible surface area (nm^2^) under the influence of an external electric field.

**Figure 9:**
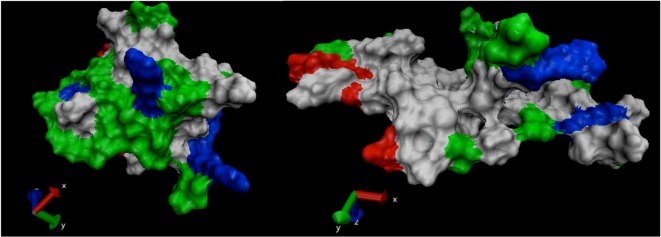
SHP snapshot of surface properties at the beginning and end of the MD simulation under electric field intensity of 3V/nm. Note: non-polar residues (white), basic (blue), acidic (red) and polar residues (green).

SASA denotes the area that is available on the protein surface for interaction with solvents and other molecules of similar sizes. Under cellular conditions, the hydrophobic residues of a protein are arranged in a manner to minimize exposure to solvents. However, when a protein is subjected to external stress such as thermal and high electric field, it undergoes conformational changes, which lead to increase in exposure of hydrophobic residues to water and other solvents. In our study, application of electric field stress of 0.002 V/nm and 0.004 V/nm had no noticeable change on SASA until the electric field intensity of 3V/nm was applied. Due to unfolding of SPI under this extreme stress, the hydrophobic and hydrophilic residues were exposed on the surface increasing the SASA (Figure 8-Figure 9). In 2011, Xiang *et al.* [8] reported that application of pulsed electric field treatment of 25 kV/cm and 120 pulses on soybean protein isolate (SPI) showed an 18% increase in surface hydrophobicity, they attributed these changes to denaturation of the protein. In the present study, the protein was subjected to external electric field in an aqueous system at neutral pH and the simulation was conducted at constant temperature and pH. Application of pulsed electric field or static high electric field to a protein in an aqueous environment would lead to generation of ohmic heating, in the study conducted by Xiang *et al.* [8], the effect of temperature was considered to be minimal. 

The findings of the present study were in accordance to several studies conducted on molecular dynamic simulation of proteins under external electric field [15,17,18]. It is important to note that the results obtained are under the assumptions of MD simulation and more analysis is required to evaluate the interaction of external field with each and every atom under the accepted force fields. Nonetheless, the MD simulation conducted for the present study does provide information about direct influence of an external electric field on the SPH. 

## 3. Experimental Section

**Figure 10:**
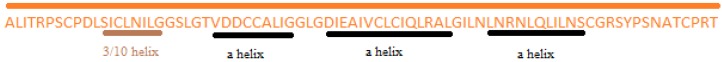
FASTA sequence of SPH protein, four disulphide bonds between Cys8-Cys43, Cys14-Cys28, Cys45-Cys77 and Cys29-Cys67 (PDB accession code 1HYP).

MD simulation on SHP was performed using a classical MD algorithm as implemented in Groningen machine for chemical simulations (GROMACS) software package, version 4.5.5 from the Stockholm Center for Biomembrane Research, Stockholm, Sweden [13]. SHP consists of 75 amino acid residues, where more than 50% of the secondary structure consists mainly of helices (4 helices; 3 - a helix (Val25-Gly33, Asp37-Leu51 and Leu56-Ser66) and 1–3/10 helix (Ser12-Gly19) (Figure 10) (PDB sequence detail). The SHP molecule surface contains 70% apolar atoms and the four disulphide bonds found in SHP provide stability to the protein (Figure 10). SHP starting configuration (PDB accession code 1HYP) was used for our study. All atom CHARMM27 force field was used to describe the potential energy and provide functions and parameters for every type of atom in the system [9]. The SHP configuration was enclosed in a periodic cubic water box at a distance of 10 Å from the edge of the box to satisfy the minimum image convention. The water model used was TIP3P [9] and one sodium ion was added to neutralize the system. The neutral solvated protein system was first energy minimized using steepest descent for 20000 steps and equilibrated at constant volume (NVT) and temperature (NPT) for 200 ps. Due to limited computing power available, the MD simulation was run for 1000 ps with temperature maintained at 300° K and using Berendsen thermostat and pressure was maintained at 1 bar. The constant temperature and pressure coupling was 0.1 ps and 2 ps respectively. To limit the short-range nonbonded interactions, van der Waals interaction and long-range electrostatic interactions, a cut-off of 1 nm was used. PME algorithm was used with grid spacing of 0.16 nm and the time step during the simulation was 2 fs [14]. MD simulations were run under constant electric field of 0.002 V/nm, 0.004 V/nm and 3 V/nm (Table 2). One MD simulation was run without electric field as a reference. The values of electric field chosen cover the achievable range of 20–40 kV/cm (0.002 V/nm and 0.004 V/nm) used commonly for pulsed electric field and electrohydrodynamic processing of food products [2]. The highest value of 3 V/nm corresponds to the values where other researchers observed structural changes in other proteins such as Chignolin [9,15]. All the electric fields were applied in the *x* axis to the equilibrated solvated protein system.

Root mean square deviation (RMSD) and radius of gyration (Rg) of the backbone atoms were calculated to represent any conformational changes in the protein. Surface hydrophobicity and hydrophylicity of the system were also estimated to evaluate the effect of electric field on the electrical and surface properties. Changes in these parameters were used as an index of protein conformational alteration and studied using GROMACS analyzing tools. The effect of external electric field on secondary structure of the protein was characterized using STRIDE algorithm implemented in visual molecular dynamics (VMD) [16]. The snapshots of protein conformational change were taken using PyMOL Molecular Graphics System, Version 1.5.0.4 Schrödinger, LLC. 

**Table 2:** Summary of parameters used in the MD simulated systems.

Protein System	Electric field strength (V/nm)	Temperature (K) and Pressure (bar)	Simulation length (ns)
SHP (1HYP)	0.002	300 K, 1 bar	1
SHP (1HYP)	0.004	300 K, 1 bar	1
SHP (1HYP)	3	300 K, 1 bar	1

## 4. Conclusions

In this work, by means of MD simulation, we explored the effect of external static electric fields of 0.002 V/nm, 0.004 V/nm and 3V/nm strength on the structural stability of soybean protein isolate. Our observation indicates that under the effect of lower intensity electric field, SHP protein reorients itself in the direction of the electric field and no major effect was observed on the secondary structure of the protein, but under 3V/nm, the protein unfolded and almost all the helical structures were lost during simulation. It can also be concluded that more work with oscillating electric field at different frequencies is required in order to estimate its effect on proteins’ structural and functional properties. Knowledge gained through MD simulation under external electric field will be useful to explain the effect of novel food processing techniques such as microwave, radiofrequency, pulsed electric field and electrohydrodynamics on the biochemical composition of food products. 
